# Theory and evidence-base for a digital platform for the delivery of language tests during awake craniotomy and collaborative brain mapping

**DOI:** 10.1136/bmjsit-2023-000234

**Published:** 2024-05-13

**Authors:** Damjan Veljanoski, Xin Yi Ng, Ciaran Scott Hill, Aimun A B Jamjoom

**Affiliations:** 1 Southwest Neurosurgery Centre, Derriford Hospital, Plymouth, UK; 2 Department of Medicine, Arrowe Park Hospital, Wirral, UK; 3 Department of Neurosurgery, National Hospital for Neurology and Neurosurgery, London, UK; 4 Centre for Clinical Brain Sciences, The University of Edinburgh, Edinburgh, UK; 5 Department of Neurosurgery, Barking Havering and Redbridge Hospitals NHS Trust, Romford, UK

**Keywords:** neurological devices, health technology, technology

## Abstract

**Objectives:**

Build the theoretical and evidence-base for a digital platform (map-OR) which delivers intraoperative language tests during awake craniotomy and facilitates collaborative sharing of brain mapping data.

**Design:**

Mixed methodology study including two scoping reviews, international survey, synthesis of development guiding principles and a risk assessment using failure modes and effects analysis.

**Setting:**

The two scoping reviews examined the literature published in the English language. International survey was completed by members of awake craniotomy teams from 14 countries.

**Main outcome measures:**

Scoping review 1: number of technologies described for language mapping during awake craniotomy. Scoping review 2: barriers and facilitators to adopting novel technology in surgery. International survey: degree of language mapping technology penetration into clinical practice.

**Results:**

A total of 12 research articles describing 6 technologies were included. The technologies required a range of hardware components including portable devices, virtual reality headsets and large integrated multiscreen stacks. The facilitators and barriers of technology adoption in surgery were extracted from 11 studies and mapped onto the 4 Unified Theory of Acceptance and Use of Technology constructs. A total of 37 awake craniotomy teams from 14 countries completed the survey. Of the responses, 20 (54.1%) delivered their language tests digitally, 10 (27.0%) delivered tests using cards and 7 (18.9%) used a combination of both. The most commonly used devices were tablet computers (67.7%; n=21) and the most common software used was Microsoft PowerPoint (60.6%; n=20). Four key risks for the proposed digital platform were identified, the highest risk being a software and internet connectivity failure during surgery.

**Conclusions:**

This work represents a rigorous and structured approach to the development of a digital platform for standardized intraoperative language testing during awake craniotomy and for collaborative sharing of brain mapping data.

**Trial registration number:**

Scoping review protocol registrations in OSF registries (scoping review 1: osf.io/su9xm; scoping review 2: osf.io/x4wsc).

**WHAT IS ALREADY KNOWN ON THIS TOPIC**

- Language mapping during awake craniotomy is the gold standard for maximizing extent of resection while limiting language deficits as a complication of brain tumor surgery; however, there is variation in the type and mode of delivery of language tests during awake craniotomies.
- Furthermore, functional language maps built using intraoperative data are limited to a small group of languages.

**WHAT THIS STUDY ADDS**

- This study lays the theory and evidence-base for the development of a digital platform for the delivery of intraoperative language tests during awake craniotomy and collaborative multilanguage brain mapping (map-OR).
- We identified a small group of technologies for the delivery of language tests during awake craniotomy.
- An international survey showed limited penetration of these technologies into clinical practice.
- Facilitators and barriers for the adoption of technology in surgery were extracted from the literature and guided key design features including using evidence-based language tests, intuitive user interface and hardware agnosticism.

**HOW THIS STUDY MIGHT AFFECT RESEARCH, PRACTICE OR POLICY**

- The work lays a robust theory and evidence-base for the development of map-OR to optimize its potential for adoption and successful implementation in both clinical and research practice.
- Map-OR aims to deliver standardized language tests during awake craniotomy, employing a range of languages.
- In conjunction, it will facilitate the mapping of positive stimulation sites onto a digital brain atlas with the ambition to prospectively build a functional multilanguage map of the human brain.
- This would serve as a resource for cognitive neuroscience research and provide invaluable insights for operative planning for neurosurgeons.

## Introduction

Human language is a complex system of communication that allows the transfer of information between individuals. It lies at the heart of building relationships, personal development and cultural transmission. Due to the central role language plays, disruption of its function negatively impacts quality of life and can shorten overall survival.^1 2^ Therefore, preserving language function is a key neurosurgical tenet for the resection of brain tumors that are adjacent to or invading regions involving critical language networks. Awake craniotomy with functional mapping using direct electrical stimulation (DES) has become the gold standard for maximizing extent of resection while limiting language deficits.^3^ Functional language mapping holds clinical value and provides an opportunity to study brain function and contribute to cognitive neuroscience. Unlike imaging modalities such as functional MRI (fMRI) which are associative, DES allows causal inference of function to brain anatomy.^4^ This has permitted the building of functional maps of human language based on intraoperative language errors during awake craniotomy.^5–8^ These maps have largely looked at single languages limiting the transferability of the findings to under-represented languages and multilingual individuals with different language combinations. Efforts have been made to retrospectively combine these maps to compare languages. Lu *et al* retrospectively created functional maps of DES-induced speech arrest and anomia by combining four large datasets that included English, French and Mandarin.^9^ The authors found a common fronto-temporo-parietal language network across the different languages which agrees with existing large-scale fMRI studies comparing languages.^10^ However, Lu *et al* also identified subtle differences such as increased speech arrest in the posterior middle frontal gyrus in the Chinese cohort compared with English and French, which is consistent with previous studies.^8^ This retrospective approach has its merits but has a number of important limitations. First, the datasets had differing language mapping strategies and stimulation intensity ranges. This variation between institutions in intraoperative language testing paradigms and interpretation is well documented.^11^ Second, the study focused on three languages (English, French and Mandarin), which only covers two language families: Indo-European (English and French) and Sino-Tibetan (Mandarin). In reality, there is huge diversity in human language with approximately 7000 languages from over 100 distinct language families spoken across the globe.^12^ This narrow view of languages is a limitation in our understanding of the neurobiology of human language. We are developing a digital platform (map-OR) for the delivery of standardized intraoperative language tests during awake craniotomy. Additionally, map-OR would facilitate annotation of a digital brain atlas to map the neuroanatomical location of language errors identified during surgery. This would allow neurosurgical teams from around the world to collaborate at scale to create a multilanguage functional map of human language. This would serve as an important contribution to cognitive neuroscience and provide invaluable insights for operative planning for neurosurgeons.

The development and translation of surgical technologies is complex. Recently, two frameworks have been described to help ensure this process is performed rigorously and in an ordered manner that balances risk and innovation. The Idea, Development, Exploration, Assessment, Long-term follow-up (IDEAL-D) collaboration proposed a model for the evaluation of device innovation.^13^ This framework included four stages: stage 1 (first in human); stage 2 (exploratory studies); stage 3 (randomized controlled trials) and stage 4 (long-term monitoring). The framework was updated to include a preclinical stage that covers analysis across four perspectives: system, device, patient and clinician.^14^ The Medical Research Council (MRC) also published guidelines on the development and evaluation of complex interventions.^15^ Complex interventions cover a wide range of interventions including welfare policy, enhanced recovery protocol and surgical procedures or devices. The framework covers four areas: development, feasibility, evaluation and implementation. One of the key differentiator of the two frameworks is the MRC’s recommendation for the use of a theory to help systematically articulate the key components of the intervention and identify uncertainties. In this article, we describe a series of mixed methodology studies (scoping reviews, international survey and risks analysis) that have been guided by both the IDEAL-D and MRC frameworks. We describe the theoretical framework being used and lay out key development guides principles. As map-OR has not been developed yet, the studies described form a preliminary IDEAL-D stage 0 focusing on systems perspective. The aim of this work is to establish a robust theoretical and evidence-based foundation for the development and adoption of map-OR.

## Methods

### Scoping review 1: what technologies have been described for language mapping during awake craniotomy?

The aim of this scoping review was to understand what technologies had been described in the literature for language mapping during awake craniotomy. A literature search was conducted in May 2022. The following electronic databases were used: MEDLINE (Ovid), Cochrane Library (Wiley), APA PsycINFO (Ovid) and Scopus. The following terms and their derivatives were used in the electronic search: ‘intra-operative’, ‘awake craniotomy’, ‘language mapping’, ‘technology’, ‘mobile’, ‘virtual reality’, ‘computer-based’ (online supplemental table 1). The inclusion criteria were any original English-language research articles or technical reports that described the use of novel software and hardware for the use of intraoperative language test delivery. Following the search, duplicate articles were removed, and one researcher (DV) then screened the titles and abstracts of articles. Two researchers (DV, AABJ) then agreed on the full articles included in the review. A range of data-points were extracted from the articles including type of technology, year it was described, country of origin, software and hardware requirements, types of language test, other cognitive tests and functionality, description of stakeholder feedback and clinical feasibility testing. The Preferred Reporting Items for Systematic Reviews and Meta-Analyses (PRISMA) scoping review checklist was used to guide this scoping review (online supplemental table 2). The scoping review protocol was published on OSF registries.^16^

10.1136/bmjsit-2023-000234.supp1 Supplementary data

### International survey examining methods of language test delivery during awake craniotomy

An international survey examining the methods used to deliver language tests during awake craniotomy was conducted between January and February 2023. The aim of the survey was to understand if awake craniotomy teams use physical cards or a digital device. If they used a digital device, what device they used and what software to deliver the tests. An online survey was developed (using Google Forms) that asked three questions: (1) How do you deliver language tests during awake craniotomies? (2) If you use a digital device, what software do you use? (3) Do you have access to the internet in your operating theatre? (online supplemental table 3). Respondents could have more than one answer per question and provided details on the country and city they worked at the start of the survey. The survey was distributed via the mailing lists of national neurosurgical societies, the personal contacts of the authors and social media platforms.

### Scoping review 2: what are the barriers and facilitators to adopting novel technology in surgery?

The aim of the second scoping review was to assess what barriers and facilitators to adopting novel technology in surgery had been described in the literature. Relevant studies were identified by electronic literature search in May 2022. The following electronic databases were used: MEDLINE (Ovid), Cochrane Library (Wiley), APA PsycINFO (Ovid) and Scopus. The following terms and their derivatives were used in the electronic search: ‘adoption’, ‘diffusion’, ‘dissemination’, ‘introduction’, ‘new’, ‘recent’, ‘innovation’, ‘novel’, ‘emergent’, ‘initial’, ‘early’, ‘preliminary’, ‘prototype’, ‘surgery’, ‘procedure’, ‘operation’, ‘device’, ‘system’, ‘imaging’, ‘application’, ‘approach’, ‘diagnostic’, ‘smartphone’, ‘mobile’, ‘software’, ‘robot’, ‘navigation’, ‘instrument’, ‘simulation’, ‘virtual’, ‘computer-based’, ‘qualitative’, ‘focus group’ (online supplemental table 4). The inclusion criteria were English-language research articles of qualitative research (interviews, discussions, questionnaires, focus groups and surveys) focusing on the adoption of novel technology in surgery. A broad definition of technology was used including both hardware and software innovations (this included but was limited to instruments, robotics, imaging, devices and software focusing on the surgical workflow). After duplicates were removed, the titles and abstracts of papers were screened (XYN). Two researchers (XYN, AABJ) conducted the final full-text screening and agreed on the included full articles. One paper was added from manual searching. The MRC guidance on the development of complex interventions recommend the use of a theory to ensure a systematic approach to developing a new intervention.^15^ Therefore, facilitators and barriers to the adoption of novel technology were extracted and mapped the four constructs of the Unified Theory of Acceptance and Use of Technology model (UTAUT).^17^ The four constructs are defined as follows: ‘performance expectancy’ refers to the degree to which an individual believes that the system will help him or her to attain gains in job performance; ‘effort expectancy’ refers to the degree of ease associated with use of the system; ‘social influence’ refers to the degree to which an individual perceives that important others believe he or she should use the new system; ‘facilitating conditions’ refers to the degree to which an individual believes that an organisation’s technical infrastructure exists to support the use of the system. The PRISMA scoping review checklist was used as guidance for this scoping review (online supplemental table 5). The scoping review protocol was published on OSF registries.^18^

### map-OR: device classification, development guiding principles and risk assessment

The core functionality of map-OR was described alongside its classification based on the IDEAL-D framework for device innovation.^14^ The insights from the two scoping reviews and international survey were distilled into a set of guiding principles for the development of map-OR. The UTAUT model was used as a framework to ensure a systematic approach was used for the synthesis of the guiding principles. Finally, a risk assessment was performed using failure modes and effects analysis (FMEA). FMEA stratifies risk based on the likelihood and severity of a particular risk. Each risk gets a rating of 1–5 for the likelihood of the risk occurring and its severity. The multiplication of these two ratings gives the stratification of risk which ranges from 1 to 25. These scores are divided into three FMEA risk categories: low (1-4), intermediate (5-9) and high (10-25).

## Results

### Scoping review 1: what technologies have been described for language mapping during awake craniotomy?

From 1292 citations, a total of 12 research articles describing 6 technologies met the inclusion criteria (online supplemental figure 1). These technologies included a personal digital assistant (PDA) app,^19^ a novel tablet platform,^20^ a virtual reality program,^21–23^ the NeuroMapper app,^24 25^ the Brain Mapping Interactive Stimulation System^8 26^ and the intraoperative examination monitor for awake surgery^27–29^ (table 1). These technologies originated from a range of countries including Japan, Canada, France, the USA and China. The technologies had substantial hardware requirements including specific devices for the delivery of language tests such as a Sony PDA, fMRI compatible tablet, virtual reality headset and dual iPads. Both the Brain Mapping Interactive Stimulation System and the intraoperative examination monitor for awake surgery included multiple integrated screens and cameras. A range of language tests were delivered using these technologies including object naming, number counting, word reading, auditory descriptive naming, famous face naming and writing tasks. Other cognitive tests included social cognition tasks using the virtual reality system.^21^ Two of the technologies reported stakeholder feedback or assessment. All 6 technologies were used in clinical practice with reported cohorts ranging from 3 to 186 patients.

**Table 1:** Technologies for the delivery of language tests during awake craniotomy

Technology	Country	Year	Summary of technology	Hardware and software requirements	Reported language tests	Other cognitive tests and functionality	Stakeholder feedback	Clinical validation
Personal digital assistant^19^	Japan	2006	Delivery of intraoperative naming tasks using PDA	Handy PDA (Sony Clie PEG−TJ25) with square (55×55 mm), back-lighted LCD and switch to facilitate changing the displayed object. Objects prepared using Microsoft PowerPoint	Object naming	None reported	None reported	3 patients
Intraoperative examination monitor for awake surgery^27–29^	Japan	2010	Intraoperative brain mapping monitor	CCD camera, 3.5-inch LCD monitor for patient and 7.5-inch LCD monitor for operator. Seven integrated parameters on the monitor: patient face and eyes, anatomical data from neuronavigation system, view of microscopic surgical field, test object, BIS, general view of operating theatre, heartbeat monitor	Object naming	Recording of patient’s face, voice, heart rate and displays BIS	Nine neurosurgeons participated in simulation using monitor technology (no patients)	2011: 220 patients across two modifications of IEMAS
Brain Mapping Interactive Stimulation System^8 26^	China	2015	Intraoperative brain mapping iintegrated task-presentation platform	Flexible and portable task presentation system including speakers to amplify patient responses, hand and foot monitoring and various interfaces (surgeon, technician, software and patient). Language tasks presented on slides projected onto a screen.	Object naming; listening comprehension; reading; number counting	Multivideo recording of patient behavior, including hand and foot monitoring. Speakers to amplify responses	None reported	2015: 66 patients 2021: 48 patients
NeuroMapper^24 25^	USA	2016	Intraoperative language and cognitive mapping using iPads	Tablet-based testing platform (NeuroMapper)—initially developed in 2016. Uses a dual iPad interface—one is patient-facing for the patient to see stimuli and the other is examiner facing for administration of the tasks	Object naming; auditory descriptive naming; famous face naming; non-word repetition; single word reading; writing	Unspecified cognitive testing	None reported	2020: 1 patient 2021: 15 patients
Novel tablet platform^20^	Canada	2016	For use during both pre-operative fMRI language mapping and DCES during awake craniotomy for glioma resection	fMRI-compatible tablet, including a touch-sensitive surface and optional stylus. Stimulus computer with flexible software (E-Prime, Psychology Software Tools) to program and execute behavioral tests, receive and quantify tablet data *Adjuncts for intraoperative use:* 5-inch LCD display (Ikan VL5), video cameras (monitoring the patient’s face, hand, foot and brain)	Number counting; word generation (verbal and written); word-copying task; tongue-movement task	Video recording of the patient’s face, hand, foot and brain	None reported	4 patients
Virtual reality^21–23^	France	2018	Delivery of intraoperative language and social cognition mapping using virtual reality technology	Interaction with an avatar using vTime app (a social network in VR). Allows creating an avatar socializing with other people in virtual environments. Hardware used was the Samsung Gear VR and Samsung S7 smartphone (android platform) with headphones. And the use of Tobii Pro VR Integration—an eye-tracking retrofitted HTC VIVE wired to a computer connected to a Brainlab neuronavigation system	Object naming (DO 80 duplicated in VRH in 2D and in stereoscope—using Unity 3D software); fluency by commenting on neuropsychologist’s avatar	Social cognition exercise; VR experience with a relaxing film at end of tumor resection during wound closure; eye tracking; tracking head orientation	Patient and medical professionals, assessing tolerance and satisfaction	2018: 3 patients 2020: 30 patients 2021: 15 patients

BIS, bispectoral index; CCD, charge coupled device; DCES, direct cortical electrical stimulation; fMRI, functional MRI; IEMAS, intraoperative examination monitor for awake surgery; LCD, liquid crystal display; MISS, Mapping Interactive Stimulation System; VR, virtual reality; VRH, virtual reality headset.

### International survey looking at practice of language mapping during awake craniotomy

**Figure 1:**
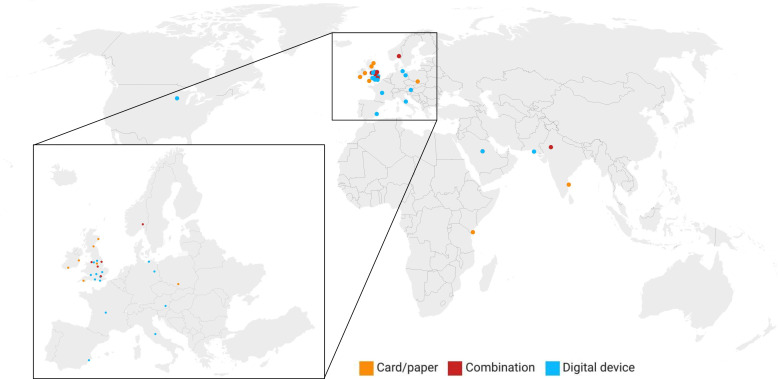
Geographical location of respondents of survey and method of language test delivery.

**Figure 2:**
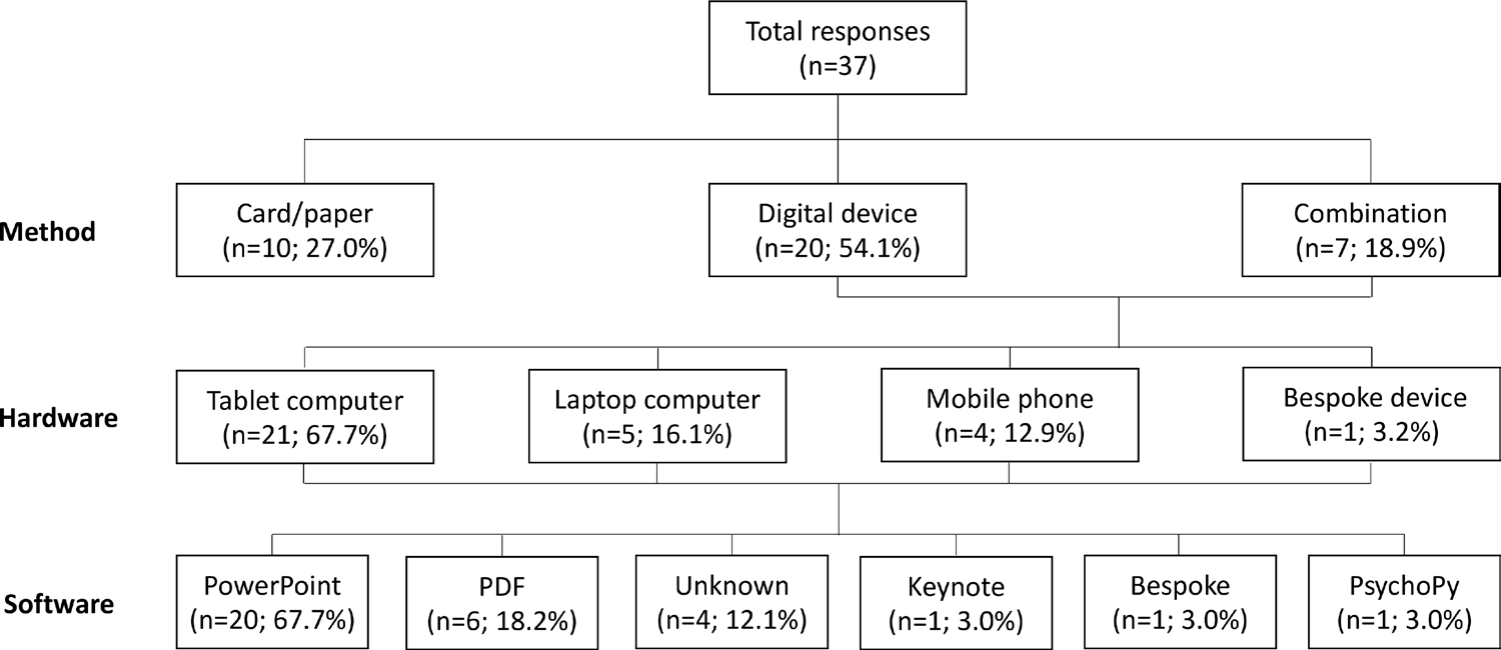
Awake craniotomy language test delivery methods from international survey including choice of hardware and software.

A total of 37 responses were captured from 14 countries (figure 1). Of the responses, 20 (54.1%) delivered their language tests digitally, 10 (27.0%) delivered tests using physical cards and 7 (18.9%) used a combination of both (figure 2). For those units that delivered their tests digitally, the most commonly used devices were tablet computers (67.7%; n=21), laptop computers (16.1%; n=5), mobile phones (12.9%; n=4) and bespoke hardware (3.2%; n=1). The predominant software used to deliver language tests was Microsoft PowerPoint (60.6%; n=20). Other software used were PDF (18.2%; n=6), Keynote (3.0%; n=1), bespoke software (3.0%; n=1) and PsychoPy (3.0%; n=1). All respondents had access to an internet connected device in their operating theatre.

### Scoping review 2: what are the barriers and facilitators to adopting novel technology in surgery?

From 1362 citations, a total of 11 research articles were included^30–40^ (online supplemental figure 2). Barriers and facilitators were mapped onto the four constructs of UTAUT model (online supplemental table 6). For the construct of ‘performance expectancy’, the main facilitators were perceived benefits to the surgeon’s technical performance and patient outcomes. ‘Performance expectancy’ barriers included beliefs that pre-existing technology functioned adequately, concerns that innovation may worsen patient outcomes and concerns about a limited evidence-base for new technology. ‘Effort expectancy’ facilitators included beliefs that the innovation would make surgery easier, shorten the learning curve and would be simple to use. Barriers in the construct of ‘effort expectancy’ focused on the impact of technical difficulties, the time and energy required for development of new skillsets and changes to the operating environment. ‘Social influence’ facilitators included patient demand, peer encouragement and approval from diverse stakeholders. On the other hand, ‘social influence’ barriers consisted of a perception that the technology was unnecessary, beliefs that adoption of novel technology would be promoted for individualistic purposes and ethical concerns about equity in access to new technology. Enabling factors for the ‘facilitating conditions’ construct included the provision of training and support, reasonable pricing and support from hospital management. Barriers to ‘facilitating conditions’ included organizational resistance to change, high costs and limited training.

### map-OR: device classification and development guiding principles

**Figure 3:**
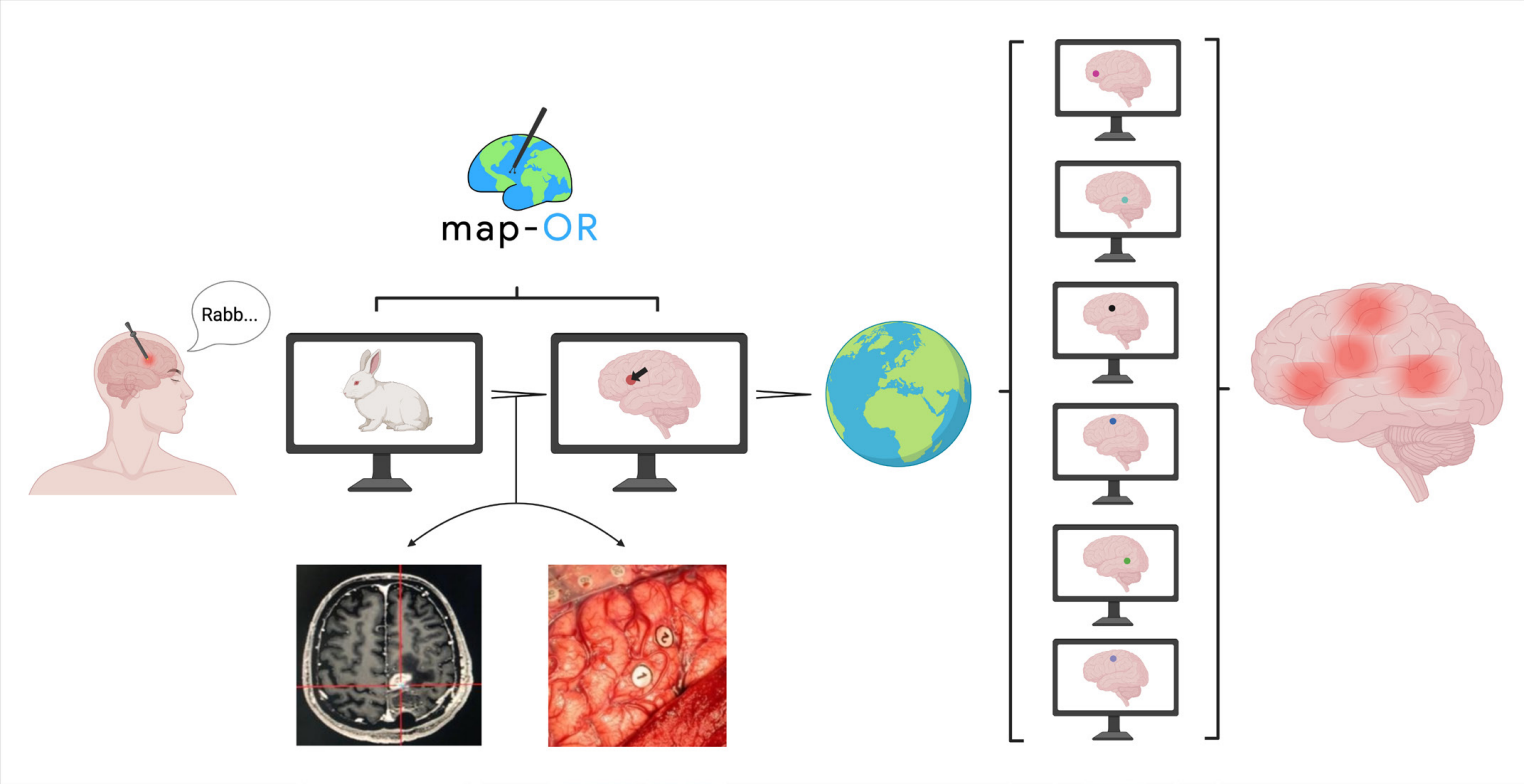
Schematic representation of map-OR’s dual functionality. Language tests are delivered via the screen of a digital device. If there is a positive stimulation then the neurosurgical team capture data on the neuroanatomical location of the stimulation site (neuronavigation localization and cortical photographs). This data are then plotted onto a digital atlas of the lateral cortex. International collaborating teams will pool these data to build a multilanguage functional map of the human brain. Created with BioRender.com.

map-OR is a piece of software that has dual functionality (figure 3). The first function is the planning and delivery of intraoperative language tests during awake craniotomy. The second function allows neurosurgeons to annotate positive stimulation sites (indicating regions of functional language localization) onto a digital atlas of the human cortex. Based on this functionality, map-OR is classified as a non-invasive and non-surgical device according to the IDEAL-D framework.^14^ Synthesizing the insights from the scoping reviews and survey, a set of development guiding principles were devised for map-OR (table 2). The UTAUT model was used as a framework to ensure the principles were developed systematically. For each UTAUT construct, a design objective was defined and onto this objective a series of map-OR features were devised to optimize its uptake. The core map-OR design objectives were to reduce pre-operative planning time through evidence-based recommendations of language tests, and to ensure enhanced intraoperative delivery efficiency. Alongside this, map-OR needs to be simple and intuitive to use. Importantly, we plan on taking a transparent approach to the development and validation of map-OR through publication in peer-reviewed journals and to build a consortium of international awake craniotomy teams to set map-OR standards through consensus. Finally, to ensure strong uptake of map-OR it must be accessible with minimal technical infrastructure.

**Table 2:** map-OR development guiding principles based on the UTAUT model

Construct	Design objective	Key map-OR feature
Performance expectancy	Reduce pre-operative planning time and enhance intraoperative delivery efficiency	Evidence-based language test recommendation based on tumor’s neuroanatomical location Evidence-based language test delivery paradigm (including recommended test delivery time and tones)
Effort expectancy	Simple intuitive design with evidence-based recommendations for language tests	Clean simple design for ease of navigation Simple interactive atlas of lateral cortex for positive stimulation site annotation Email notifications to map positive stimulation sites onto brain atlas
Social influence	Build a community working towards standardizing language tests during awake craniotomy and goal to build a multilanguage map of the human brain using direct electrical stimulation	Use a transparent and evidence-based approach to the development and validation of map-OR with a strong focus on publication in peer-reviewed journals Set up a consortium of like-minded neurosurgical teams around the world to set language testing and atlas mapping standards through consensus
Facilitating conditions	Accessible with minimal technical infrastructure	map-OR to be delivered as a web application that is accessible through standard web browser Hardware agnostic

UTAUT, Unified Theory of Acceptance and Use of Technology.

### Risk assessment using failure modes and effects analysis

As recommended by IDEAL, we used the FMEA framework to conduct a systematic risk assessment. We identified four key risks attached to map-OR (table 3). The risk with the highest risk stratification was software or internet connection failure with an intermediate FMEA risk category. The possible side effect of this risk was disruption or delay to the language test delivery which could compromise a safe awake craniotomy. This risk could be mitigated by improved software testing and having a cache of language test material held on local device storage. The second risk identified was the recommendation of incorrect language tests which was deemed an intermediate FMEA risk category. Approaches to reduce the risk of this was to ensure building cross-checking for test recommendation and comprehensive user training. The third risk identified was patient data breaches which had a low FMEA risk category. To address this risk, map-OR should have industry standard encryption and require user identification. The final risk was incorrect annotation of the digital brain atlas which was deemed to be a low FMEA risk category. To manage this risk, users would get comprehensive training and each annotation would undergo consensus validation.

**Table 3:** map-OR risk assessment using FMEA

Failure mode	Possible effect	Likelihood (rating)	Severity (rating)	Stratification of risk (FMEA risk category)	Mitigation plan
Software failure during procedure (including loss of internet connectivity)	Disruption or delay to surgery. Inability to deliver language tests leading to less safe surgery	Occasional: <1/100 (3)	Serious (3)	9 (intermediate)	Improve software testing procedures. Improve error handling for file corruptions. Cache language test materials on local device storage.
Incorrect language test recommendation or delivery	Erroneous surgical decision-making leading to iatrogenic language deficit	Remote: <1/1000 (2)	Serious (4)	8 (intermediate)	Build in cross-checking of test recommendations. Consensus validation of language test choice. Comprehensive user manuals and training.
Patient data breach	Serious breach of patient confidentiality. Could undermine trust in the system	Remote: <1/1000 (2)	Minor (2)	4 (low)	Use encryption methods for all transfer and storage of patient data. Require user authentication for access. Security training for users.
Incorrect brain atlas annotation	Incorrect annotation of stimulation sites invalidating scientific value of atlas	Occasional: <1/100 (3)	Negligible (1)	3 (low)	Comprehensive user manuals and training. Build in cross-checking of data entry and annotations. Use consensus validation of language maps.

FMEA, failure modes and effects analysis.

## Discussion

In this article, we describe the theory and evidence-base for developing a digital platform (map-OR) for the standardized delivery of intraoperative language tests during awake craniotomy, and to also facilitate collaborative sharing of brain mapping data. The driving force behind this work is to address two issues. The first is a lack of standardization in the delivery of language tests during awake craniotomy. This lack of standardization is at multiple levels encompassing the type of tests, the images used, the way they are delivered and how errors are interpreted. A review by Papatzalas *et al* found that a significant number of reported series used mixed or homemade test batteries.^41^ A survey of 137 specialists (including neurosurgeons, neuropsychologists and speech therapists) found high inter-rater variation in choice of language tests and interpretation of errors.^11^ The second issue is the bias in the language maps which have been described in the literature. The predominant language maps have been in English, French and Mandarin.^9^ If we consider native speakers, this constitutes approximately 17.5% (1.4 out of 8 billion) of the global population.^12^ This bias limits the generalizability of the maps and comparisons between languages. As shown by Lu *et al*, DES has identified differences in functional neuroanatomy between Mandarin and English/French.^9^ As such, map-OR has two aims. First, to deliver standardized language tests during awake craniotomy covering a range of languages. Second, to facilitate the mapping of positive stimulation sites onto a digital brain atlas with the ambition to prospectively build a functional multilanguage map of the human brain. This would serve as a resource for cognitive neuroscience research and provide invaluable insights for operative neurosurgical planning.

To ensure a rigorous approach, we used a combination of the MRC framework for complex interventions and the IDEAL-D framework for surgical innovation.^14 15^ The updated MRC framework recommends using a theory to ensure a systematic approach to developing a new intervention. In the case of map-OR, we were particularly interested in developing a technology that would be accepted and used by awake craniotomy teams. We therefore used the UTAUT model to provide a theoretical framework for developing map-OR.^17^ UTAUT provided a structured framework to examine the facilitators and barriers to technology adoption by surgical teams. In conjunction, it ensured thorough and robust considerations when developing the guiding principles for map-OR. We also used the preclinical phase of the IDEAL-D framework for device innovation.^14^ IDEAL-D classifies four types of preclinical studies for device innovation: (1) device perspective studies which examine technical effectiveness and safety profile; (2) patient acceptability studies which would involve patient and public involvement; (3) clinician perspective studies which examine clinician usability and preferences and (4) system-level studies which explore gaps within the evidence and current healthcare systems. Typically, IDEAL-D stage 0 analysis occurs after the development of the innovation to allow feedback from these various perspectives. In this article, we present preliminary IDEAL-D stage 0 work which has occurred prior to starting the development of map-OR. We focused on system-level studies (two scoping reviews and international survey) to build the rationale for map-OR and inform its development. Prior to building the technical infrastructure of map-OR, there are a number of outstanding questions to address including which language tests should be used, how to define language errors and the choice of brain atlas. We plan on building a collaboration of international awake craniotomy teams and come to agreement on these questions using an expert Delphi consensus process.

Our scoping review of language mapping technologies used during awake craniotomy found a limited range of six technologies described in the literature. These technologies ranged from a simple PDA application which showed images for picture naming through to large integrated multiscreen platforms with a breadth of functionality. What was particularly striking was that all the technologies described had specific hardware requirements. This limits the dissemination and uptake of these technologies as other awake craniotomy teams need to invest in the hardware. This was evident in the international survey where none of the respondents used any of the technologies that had been described in the literature. Approximately three-quarters of respondents delivered language tests using a digital device of which 9 out of 10 of the pieces of software used were proprietary (PowerPoint, Keynote and PDF) and were not designed for language test delivery. The widespread use of software that is not designed specifically for intraoperative language testing will further increase the variation in test delivery. For example, language mapping protocols recommend an auditory cue to indicate to the surgical team when a new image has been shown to the patient which should be visible for 4 s during which time DES is applied.^42 43^ This functionality is not available or easily set up using the range of software used by the survey respondents.

To address the issue of limited technology adoption, we took a systematic approach to setting our map-OR’s development guiding principles. We laid out four key design objectives guided by UTAUT covering the four constructs: performance expectancy, effort expectancy, social influence and facilitating conditions. One of the key features that emerged from this process was the importance of using evidence-based language tests agreed through consensus using a transparent process. There are a number of published protocols in the literature for language testing during awake craniotomy.^42 44–46^ As our ambition is to create a multilanguage functional map of the human brain, it will be vital to ensure the language test paradigms is validated for a range of languages such as the MULTIMAP picture naming test.^47^ The map-OR feature that emerged from the other two UTAUT constructs (effort expectancy and facilitating conditions) centred on ensuring ease of use of map-OR through an intuitive user interface and minimal hardware requirements. The international survey highlighted the range of different devices used by awake craniotomy teams. Therefore, map-OR will be delivered as a web application to allow it to be accessible on any internet connected device.

The work presented in this article has a number of limitations. First, the international survey had a small number of responses which were mainly located in Europe. This needs to be considered when interpreting the results as they are unlikely to be representative, particularly for teams working in North America. Second, the work in this article is preliminary IDEAL-D stage 0 as development of map-OR has not been completed. This analysis focused solely on system perspectives and did not include patient or clinician views. We are planning to complete IDEAL-D stage 0 after the development of minimal viable product of map-OR.

In conclusion, our study presents a rigorous, structured approach to the development of a digital platform for standardized intraoperative language testing during awake craniotomy and collaborative brain mapping. The work lays a robust theory and evidence-base for the development of map-OR to give it the best chance of adoption and successful implementation. By leveraging the power of collaboration, map-OR holds the potential to facilitate the creation of a multilanguage functional map of human language, contributing significantly to the field of cognitive neuroscience.

## Data Availability

Data are available upon reasonable request.

## References

[1] Hilari K , Needle JJ , Harrison KL . What are the important factors in health-related quality of life for people with aphasia? A systematic review. Arch Phys Med Rehabil 2012;93:S86–95. 10.1016/j.apmr.2011.05.028 22119074

[2] McGirt MJ , Mukherjee D , Chaichana KL , et al . Association of surgically acquired motor and language deficits on overall survival after resection of glioblastoma multiforme. Neurosurgery 2009;65:463–70. 10.1227/01.NEU.0000349763.42238.E9 19687690

[3] De Witt Hamer PC , Robles SG , Zwinderman AH , et al . Impact of intraoperative stimulation brain mapping on glioma surgery outcome: a meta-analysis. JCO 2012;30:2559–65. 10.1200/JCO.2011.38.4818 22529254

[4] Siddiqi SH , Kording KP , Parvizi J , et al . Causal mapping of human brain function. Nat Rev Neurosci 2022;23:361–75. 10.1038/s41583-022-00583-8 35444305 PMC9387758

[5] Chang EF , Breshears JD , Raygor KP , et al . Stereotactic probability and variability of speech arrest and Anomia sites during stimulation mapping of the language dominant hemisphere. JNS 2017;126:114–21. 10.3171/2015.10.JNS151087 26894457

[6] Tate MC , Herbet G , Moritz-Gasser S , et al . Probabilistic map of critical functional regions of the human cerebral cortex: Broca’s area Revisited. Brain 2014;137:2773–82. 10.1093/brain/awu168 24970097

[7] Sanai N , Mirzadeh Z , Berger MS . Functional outcome after language mapping for glioma resection. N Engl J Med 2008;358:18–27. 10.1056/NEJMoa067819 18172171

[8] Wu J , Lu J , Zhang H , et al . Direct evidence from intraoperative electrocortical stimulation indicates shared and distinct speech production center between Chinese and English languages. Hum Brain Mapp 2015;36:4972–85. 10.1002/hbm.22991 26351094 PMC6869327

[9] Lu J , Zhao Z , Zhang J , et al . Functional maps of direct electrical stimulation-induced speech arrest and anomia: a multicentre retrospective study. Brain 2021;144:2541–53. 10.1093/brain/awab125 33792674 PMC8453410

[10] Malik-Moraleda S , Ayyash D , Gallée J , et al . An investigation across 45 languages and 12 language families reveals a universal language network. Nat Neurosci 2022;25:1014–9. 10.1038/s41593-022-01114-5 35856094 PMC10414179

[11] Sefcikova V , Sporrer JK , Ekert JO , et al . High Interrater variability in intraoperative language testing and interpretation in awake brain mapping among neurosurgeons or neuropsychologists: an emerging need for standardization. World Neurosurgery 2020;141:e651–60. 10.1016/j.wneu.2020.05.250 32522656

[12] Lewis MP . Ethnologue: languages of the world. SIL Int, 2009.

[13] Hirst A , Philippou Y , Blazeby J , et al . No surgical innovation without evaluation: evolution and further development of the IDEAL framework and recommendations. Ann Surg 2019;269:211–20. 10.1097/SLA.0000000000002794 29697448

[14] Marcus HJ , Bennett A , Chari A , et al . IDEAL-D framework for device innovation: a consensus statement on the preclinical stage. Ann Surg 2022;275:73–9. 10.1097/SLA.0000000000004907 33856386 PMC8683254

[15] Skivington K , Matthews L , Simpson SA , et al . A new framework for developing and evaluating complex interventions: update of medical research Council guidance. BMJ 2021;374:n2061. 10.1136/bmj.n2061 34593508 PMC8482308

[16] Jamjoom A . What technologies have been described for language mapping during awake craniotomy? OSF, 2022. Available: https://osf.io/su9xm

[17] Venkatesh V , Morris MG , Davis GB , et al . User acceptance of information technology: toward a unified view. MIS Quarterly 2003;27:425. 10.2307/30036540

[18] Jamjoom A . Barriers and facilitators to adopting novel technology in surgery. OSF Registries. OSF, 2022. Available: https://osf.io/x4wsc

[19] Sato S , Oka H , Utsuki S , et al . Utilization of personal digital assistants (PDA) for intraoperative naming tasks in awake surgery. Minim Invasive Neurosurg 2006;49:58–9. 10.1055/s-2005-919166 16547885

[20] Morrison MA , Tam F , Garavaglia MM , et al . A novel tablet computer platform for advanced language mapping during awake craniotomy procedures. JNS 2016;124:938–44. 10.3171/2015.4.JNS15312 26473779

[21] Bernard F , Lemée J-M , Aubin G , et al . Using a virtual reality social network during awake craniotomy to map social cognition: prospective trial. J Med Internet Res 2018;20:e10332. 10.2196/10332 29945859 PMC6039768

[22] Delion M , Klinger E , Bernard F , et al . Immersing patients in a virtual reality environment for brain mapping during awake surgery: safety study. World Neurosurg 2020;134:e937–43. 10.1016/j.wneu.2019.11.047 31734424

[23] Casanova M , Clavreul A , Soulard G , et al . Immersive virtual reality and ocular tracking for brain mapping during awake surgery: prospective evaluation study. J Med Internet Res 2021;23:e24373. 10.2196/24373 33759794 PMC8074984

[24] Sabsevitz DS , Middlebrooks EH , Tatum W , et al . Examining the function of the visual word form area with stereo EEG electrical stimulation: a case report of pure Alexia. Cortex 2020;129:112–8. 10.1016/j.cortex.2020.04.012 32442776

[25] Suarez-meade P , Marenco-hillembrand L , Sabsevitz D , et al . Preprint: time to re-think Broca: extent of resection and neurological outcome in patients harboring tumors in the dominant inferior frontal gyrus. 2024.

[26] Hameed NUF , Zhao Z , Zhang J , et al . A novel intraoperative brain mapping integrated task-presentation platform. Operative Surg 2021;20:477–83. 10.1093/ons/opaa476 33548926

[27] Yoshimitsu K , Suzuki T , Muragaki Y , et al . Development of modified intraoperative examination monitor for awake surgery (IEMAS) system for awake craniotomy during brain tumor resection. Annu Int Conf IEEE Eng Med Biol Soc 2010;2010:6050–3. 10.1109/IEMBS.2010.5627616 21097121

[28] Yoshimitsu K , Maruyama T , Muragaki Y , et al . Wireless modification of the intraoperative examination monitor for awake surgery. Neurol Med Chir (Tokyo) 2011;51:472–6. 10.2176/nmc.51.472 21701117

[29] Fukutomi Y , Yoshimitsu K , Tamura M , et al . Quantitative evaluation of efficacy of intraoperative examination monitor for awake surgery. World Neurosurg 2019;126:e432–8. 10.1016/j.wneu.2019.02.069 30825617

[30] Seyed Esfahani M , Heydari Khajehpour S , Roushan-Easton G , et al . A framework for successful adoption of surgical innovation. Surg Innov 2022;29:662–70. 10.1177/15533506221074612 35315708 PMC9615345

[31] Benmessaoud C , Kharrazi H , MacDorman KF . Facilitators and barriers to adopting robotic-assisted surgery: contextualizing the unified theory of acceptance and use of technology. PLoS One 2011;6:e16395. 10.1371/journal.pone.0016395 21283719 PMC3024425

[32] Krishnan G , Mintz J , Foreman A , et al . The acceptance and adoption of transoral robotic surgery in Australia and New Zealand. J Robotic Surg 2019;13:301–7. 10.1007/s11701-018-0856-8 30128930

[33] Goncąlves AA , Castro Silva SL , Pitassi C , et al . Innovation in cancer treatment: Theimpacts of Robotic-assisted surgery adoption at the Brazilian National Cancer Institute. In: Studies in health technology and informatics. 2020: 123–6. Available: https://www.scopus.com/inward/record.uri?eid=2-s2.0-85087405233&doi=10.3233%2FSHTI200509&partnerID=40&md5=cdae83bd9be642777b777ecd1a95643c 10.3233/SHTI200509 32604616

[34] Jaiprakash A , O’Callaghan WB , Whitehouse SL , et al . Orthopaedic surgeon attitudes towards current limitations and the potential for robotic and technological innovation in arthroscopic surgery. J Orthop Surg (Hong Kong) 2017;25:2309499016684993. 10.1177/2309499016684993 28142353

[35] Hsu H-M , Chang I-C , Lai T-W . Physicians’ perspectives of adopting computer-assisted navigation in orthopedic surgery. Int J Med Inform 2016;94:207–14. 10.1016/j.ijmedinf.2016.07.006 27573328

[36] Abrishami P , Boer A , Horstman K . Understanding the adoption dynamics of medical innovations: affordances of the DA Vinci robot in the Netherlands. Soc Sci Med 2014;117:125–33. 10.1016/j.socscimed.2014.07.046 25063968

[37] Felgner S , Ex P , Henschke C . Physicians’ decision making on adoption of new technologies and role of coverage with evidence development: a qualitative study. Value Health 2018;21:1069–76. 10.1016/j.jval.2018.03.006 30224111

[38] Abrishami P , Boer A , Horstman K . When the evidence basis breeds controversies: exploring the value profile of robotic surgery beyond the early introduction phase. Med Care Res Rev 2020;77:596–608. 10.1177/1077558719832797 30902036

[39] Cundy TP , Marcus HJ , Hughes-Hallett A , et al . International attitudes of early adopters to current and future robotic technologies in pediatric surgery. Journal of Pediatric Surgery 2014;49:1522–6. 10.1016/j.jpedsurg.2014.05.017 25280660

[40] Catchpole KR , Hallett E , Curtis S , et al . Diagnosing barriers to safety and efficiency in robotic surgery. Ergonomics 2018;61:26–39. 10.1080/00140139.2017.1298845 28271956 PMC6010349

[41] Papatzalas C , Fountas K , Kapsalaki E , et al . The use of standardized intraoperative language tests in awake craniotomies: a scoping review. Neuropsychol Rev 2022;32:20–50. 10.1007/s11065-021-09492-6 33786797

[42] Gogos AJ , Young JS , Morshed RA , et al . Awake glioma surgery: technical evolution and nuances. J Neurooncol 2020;147:515–24. 10.1007/s11060-020-03482-z 32270374

[43] Hervey-Jumper SL , Li J , Lau D , et al . Awake craniotomy to maximize glioma resection: methods and technical nuances over a 27-year period. JNS 2015;123:325–39. 10.3171/2014.10.JNS141520 25909573

[44] De Witte E , Satoer D , Robert E , et al . The Dutch linguistic intraoperative protocol: a valid linguistic approach to awake brain surgery. Brain Lang 2015;140:35–48. 10.1016/j.bandl.2014.10.011 25526520

[45] Rosengarth K , Pai D , Dodoo-Schittko F , et al . A novel language paradigm for intraoperative language mapping: feasibility and evaluation. J Clin Med 2021;10:655. 10.3390/jcm10040655 33567742 PMC7915060

[46] Połczyńska M . New tests for language mapping with intraoperative electrical stimulation of the brain to preserve language in individuals with tumors and epilepsy: a preliminary follow-up study. Poznań Studies in Contemporary Linguistics 2009;45:261–79. 10.2478/v10010-009-0015-5

[47] Gisbert-Muñoz S , Quiñones I , Amoruso L , et al . MULTIMAP: multilingual picture naming test for mapping eloquent areas during awake surgeries. Behav Res Methods 2021;53:918–27. 10.3758/s13428-020-01467-4 32901346 PMC8062318

