# Supplementary material for: Theory and evidence-base for a digital platform for the delivery of language tests during awake craniotomy and collaborative brain mapping
Source: BMJ Surg Interv Health Technol. 2024 May 13;6(1):e000234. doi: 10.1136/bmjsit-2023-000234 (PMC11097893; doi:10.1136/bmjsit-2023-000234)
Supplement: Supplementary data [file bmjsit-2023-000234supp001.pdf]

SUPPLEMENTARY MATERIAL

Supplementary material for *"Development of a digital platform for the delivery of intraoperative language tests during awake craniotomy and for collaborative brain mapping (IDEAL Stage 0)"*

Supplementary material	Pages
**Supplementary Table 1:** Search strategy for scoping review 1 "What technologies have been described for language mapping during awake craniotomy?"	2-3
**Supplementary table 2**: Preferred Reporting Items for Systematic reviews and Meta-Analyses extension for Scoping Reviews (PRISMA-ScR) Checklist (Scoping review 1)	4-5
**Supplementary table 3**: International survey examining methods of language test delivery during awake craniotomy	6
**Supplementary Table 4:** Search strategy for scoping review 2 "What are the barriers and facilitators to adopting novel technology in surgery?"	7-9
**Supplementary table 5**: Preferred Reporting Items for Systematic reviews and Meta-Analyses extension for Scoping Reviews (PRISMA-ScR) Checklist (Scoping review 2)	10-11
**Supplementary Figure 1**: PRISMA flowchart for Scoping review 1 "What technologies have been described for language mapping during awake craniotomy?"	12
**Supplementary Figure 2:** PRISMA flowchart for scoping review 2: "What are the barriers and facilitators to adopting novel technology in surgery?"	13
**Supplementary Table 6**: barriers and facilitators to adoption of novel technology by surgical teams mapped onto the UTAUT constructs	14-17

Supplementary Table 1: Search strategy for scoping review 1 "What technologies have been described for language mapping during awake craniotomy?"

Database	Search strategy	Hits
MEDLINE(Ovid)<br>✓	1) ("intraoperative" or "craniotom*" or "awake craniotom*").ti,ab. or exp Craniotomy/<br>2) "language mapping".ti,ab. or exp Brain Mapping/ or exp Language tests/<br>3) exp Technology/ or ("device*" or "imaging" or "remote" or "smartphone" or "mobile" or "eHealth" or "robot*" or "navigation" or "instrument*" or "simulation*" or "virtual" or "virtual reality" or "computer-based" or "computer based").ti,ab.<br>4) 1 and 2 and 3	MEDLINE: 572
Cochrane Library (Wiley)<br>✓	1) #1 ("intraoperative" or "craniotom*" or "awake craniotom*"):ti,ab<br>2) #2 MeSH descriptor: [Craniotomy] explode all trees<br>3) #3 #1 or #2<br>4) #4 MeSH descriptor: [Brain Mapping] explode all trees<br>5) #5 MeSH descriptor: [Language Tests] explode all trees<br>6) #6 "language mapping":ti,ab<br>7) #7 #4 or #5 or #6<br>8) #8 MeSH descriptor: [Technology] explode all trees<br>9) #9 ("device*" or "imaging" or "remote" or "smartphone" or "mobile" or "eHealth" or "robot*" or "navigation" or "instrument*" or "simulation*" or "virtual" or "virtual reality" or "computer-based" or "computer based"):ti,ab<br>10) #10 #8 or #9<br>11) #11 #3 and #7 and #10	6
APA PsycInfo (Ovid)<br>✓	1) ("craniotom*" or "awake craniotom*").ti,ab,mp.<br>2) ("language mapping" or "brain mapping" or "test*" or "language" or "intraoperative").ti,ab,mp. or exp Stereotaxic Atlas/<br>3) exp Technology/ or ("device*" or "imaging" or "remote" or "smartphone" or "mobile" or "eHealth" or "robot*" or "navigation" or "instrument*" or "simulation*" or "virtual" or "virtual reality" or "computer-based" or "computer based").ti,ab.<br>1 and 2 and 3	47
Scopus	(TITLE-ABS ("craniotom*" OR "awake craniotom*") AND TITLE-ABS ("language mapping" OR "brain mapping" OR "language test*" OR "intraoperative") AND TITLE-ABS ("technolog*" OR "device*" OR "imaging" OR "remote" OR "smartphone" OR "mobile" OR	661

	"eHealth" OR "robot*" OR "navigation" OR "instrument*" OR "simulation*" OR "virtual" OR "virtual reality" OR "computer-based" OR "computer based")) AND (LIMIT-TO (SUBJAREA , "MEDI")) AND (LIMIT-TO (LANGUAGE , "English"))	

Supplementary table 2: Preferred Reporting Items for Systematic reviews and Meta-Analyses extension for Scoping Reviews (PRISMA-ScR) Checklist (Scoping review 1)

SECTION	ITEM	PRISMA-ScR CHECKLIST ITEM	REPORTED ON PAGE #
**TITLE**			
Title	1	Identify the report as a scoping review.	na
**ABSTRACT**			
Structured summary	2	Provide a structured summary that includes (as applicable): background, objectives, eligibility criteria, sources of evidence, charting methods, results, and conclusions that relate to the review questions and objectives.	2-3
**INTRODUCTION**			
Rationale	3	Describe the rationale for the review in the context of what is already known. Explain why the review questions/objectives lend themselves to a scoping review approach.	5-6
Objectives	4	Provide an explicit statement of the questions and objectives being addressed with reference to their key elements (e.g., population or participants, concepts, and context) or other relevant key elements used to conceptualize the review questions and/or objectives.	7
**METHODS**			
Protocol and registration	5	Indicate whether a review protocol exists; state if and where it can be accessed (e.g., a Web address); and if available, provide registration information, including the registration number.	7
Eligibility criteria	6	Specify characteristics of the sources of evidence used as eligibility criteria (e.g., years considered, language, and publication status), and provide a rationale.	7
Information sources*	7	Describe all information sources in the search (e.g., databases with dates of coverage and contact with authors to identify additional sources), as well as the date the most recent search was executed.	7
Search	8	Present the full electronic search strategy for at least 1 database, including any limits used, such that it could be repeated.	Supp page 2-3
Selection of sources of evidence†	9	State the process for selecting sources of evidence (i.e., screening and eligibility) included in the scoping review.	7
Data charting process‡	10	Describe the methods of charting data from the included sources of evidence (e.g., calibrated forms or forms that have been tested by the team before their use, and whether data charting was done independently or in duplicate) and any processes for obtaining and confirming data from investigators.	7
Data items	11	List and define all variables for which data were sought and any assumptions and simplifications made.	7
Critical appraisal of individual sources of evidence§	12	If done, provide a rationale for conducting a critical appraisal of included sources of evidence; describe the methods used and how this information was used in any data synthesis (if appropriate).	n/a
Synthesis of results	13	Describe the methods of handling and summarizing the data that were charted.	7
**RESULTS**			

SECTION	ITEM	PRISMA-ScR CHECKLIST ITEM	REPORTED ON PAGE #
Selection of sources of evidence	14	Give numbers of sources of evidence screened, assessed for eligibility, and included in the review, with reasons for exclusions at each stage, ideally using a flow diagram.	Supp page 12
Characteristics of sources of evidence	15	For each source of evidence, present characteristics for which data were charted and provide the citations.	10-13
Critical appraisal within sources of evidence	16	If done, present data on critical appraisal of included sources of evidence (see item 12).	n/a
Results of individual sources of evidence	17	For each included source of evidence, present the relevant data that were charted that relate to the review questions and objectives.	10-13
Synthesis of results	18	Summarize and/or present the charting results as they relate to the review questions and objectives.	10
**DISCUSSION**			
Summary of evidence	19	Summarize the main results (including an overview of concepts, themes, and types of evidence available), link to the review questions and objectives, and consider the relevance to key groups.	19
Limitations	20	Discuss the limitations of the scoping review process.	20
Conclusions	21	Provide a general interpretation of the results with respect to the review questions and objectives, as well as potential implications and/or next steps.	20
**FUNDING**			
Funding	22	Describe sources of funding for the included sources of evidence, as well as sources of funding for the scoping review. Describe the role of the funders of the scoping review.	21

Supplementary table 3: International survey examining methods of language test delivery during awake craniotomy

Question	Response
Which city and country do you work in?	*Free text*
Which hospital do you work in?	*Free text*
How do you deliver your language tests to patients during an awake craniotomy?	Card/paper
	Desktop computer
	Laptop computer
	Tablet computer
	Mobile phone
	Other
If you use a digital device, what software do you use?	PowerPoint (Microsoft)
	Keynote (Apple)
	Neuro-mapper app
	Neuroons presentation
	PDF
	Other
Do you have access to an internet connected device in your operating rooms?	Yes
	No

Veljanoski D, *et al. BMJ Surg Interv Health Technologies* 2024; 6:e000234. doi: 10.1136/bmjsit-2023-000234

Supplementary Table 4: Search strategy for scoping review 2 "What are the barriers and facilitators to adopting novel technology in surgery?"

Database	Search strategy	Hits
MEDLINE(Ovid) and Cochrane Library (Wiley) ✓	1) (introduc* or adopt*).ti,ab. adj5 (exp "Diffusion of Innovation"/ or ("new" or "newly" or "recent*" or "innovat*" or "novel" or "emergent" or "early" or "initial" or "preliminary" or "prototype").ti,ab.) adj5 (exp Technology/ or ("device*" or "imaging" or "remote" or "smartphone" or "mobile" or "eHealth" or "robot*" or "navigation" or "simulation*" or "virtual" or "virtual reality" or "computer-based" or "computer based").ti,ab.)<br>2) ("surgery" or "surgeries" or "surgical").ti,ab. or exp General Surgery/ or exp Surgical Procedures, Operative/<br>3) (("semi-structured" or semistructured or unstructured or informal or "in-depth" or "face-to-face" or structured or guide) adj3 (interview* or discussion* or questionnaire* or survey*)).ti,ab.<br>4) exp Interviews as Topic/mt or exp Focus Groups/mt or exp Narration/ or exp Qualitative Research/ or qualitative.mp.<br>5) (view* or perspective* or opinion*).mp.<br>6) exp Attitude of Health Personnel/<br>7) 3 or 4 or 5 or 6<br>8) 1 and 2 and 7	MEDLINE: 195
Cochrane lib ✓	#1 MeSH descriptor: [Diffusion of Innovation] explode all trees<br>#2 ("new" or "newly" or "recent*" or "innovat*" or "novel" or "emergent" or "early" or "initial" or "preliminary" or "prototype"):ti,ab<br>#3 #1 or #2<br>#4 MeSH descriptor: [Technology] explode all trees<br>#5 ("device*" or "imaging" or "remote" or "smartphone" or "mobile" or "eHealth" or "robot*" or "navigation" or "instrument*" or "simulation*" or "virtual" or "virtual reality" or "computer-based" or "computer based"):ti,ab<br>#6 #4 or #5<br>#7 #3 adj5 #6<br>#8 MeSH descriptor: [Interviews as Topic] explode all trees<br>#9 MeSH descriptor: [Focus Groups] explode all trees<br>#10 MeSH descriptor: [Narration] explode all trees<br>#11 MeSH descriptor: [Qualitative Research] explode all trees	13

Veljanoski D, *et al. BMJ Surg Interv Health Technologies* 2024; 6:e000234. doi: 10.1136/bmjsit-2023-000234

	#12 qualitative.mp<br>#13 (("semi-structured" or semistructured or unstructured or informal or "in-depth" or "face-to-face" or structured or guide) adj3 (interview* or discussion* or questionnaire* or survey*)):ti,ab<br>#14 (view* or perspective* or opinion*).mp.<br>#15 MeSH descriptor: [Attitude of Health Personnel] explode all trees<br>#16 #8 or #9 or #10 or #11 or #12 or #13 or #14 or #15<br>#17 ("surgery" or "surgeries" or "surgical"):ti,ab<br><br>#18 MeSH descriptor: [General Surgery] explode all trees<br>#19 MeSH descriptor: [Surgical Procedures, Operative] explode all trees<br>#20 #17 or #18 or #19<br>#21 (introduc* or adopt*):ti,ab<br>#22 #21 adj5 #7<br>#23 #22 and #16 and #20	
APA PsycInfo (Ovid)<br><br>✓	1) (introduc* or adopt*).ti,ab. adj5 (exp Innovation/ or ("new" or "newly" or "recent*" or "innovat*" or "novel" or "emergent" or "early" or "initial" or "preliminary" or "prototype").ti,ab.) adj5 (exp Technology/ or exp Computer applications/ or ("device*" or "imaging" or "remote" or "smartphone" or "mobile" or "eHealth" or "robot*" or "navigation" or "simulation*" or "virtual" or "virtual reality" or "computer-based" or "computer based").ti,ab.)<br>2) ("surgery" or "surgeries" or "surgical").ti,ab. or exp Surgery/<br>3) (("semi-structured" or semistructured or unstructured or informal or "in-depth" or indepth or "face-to-face" or structured or guide or guides) adj3 (interview* or discussion* or questionnaire*)).ti,ab,id.<br>4) (focus group* or qualitative or ethnograph* or fieldwork or "field work" or "key informant").ti,ab,id.<br>5) qualitative research/ or interviews/ or group discussion/ or qualitative study.md. or experiences.tw. or interview.tw. or qualitative.tw.<br>6) 3 or 4 or 5<br>7) 1 and 2 and 6	10
Scopus	(TITLE(("introduce*" OR "new" OR "newly" OR "recent*" OR "innovat*" OR "novel" OR "emergent" OR "early" OR "initial" OR "preliminary" OR "prototype"))<br>AND TITLE(("technolog*" OR "imaging" OR "remote" OR "smartphone" OR "mobile" OR "eHealth" OR "robot*" OR "simulation*" OR "virtual" OR "virtual reality" OR "computer-based" OR "computer based"))	1144

Veljanoski D, et al. BMJ Surg Interv Health Technologies 2024; 6:e000234. doi: 10.1136/bmjsit-2023-000234

	AND TITLE-ABS-KEY(("surgery" OR "surgeries" OR "surgical"))<br>AND TITLE-ABS-KEY((opinion* OR informal OR "in-depth" OR guide* OR interview* OR discussion* OR questionnaire* OR "focus group*" OR qualitative)))<br>AND (LIMIT-TO (PUBSTAGE,"final"))<br>AND (LIMIT-TO (SUBJAREA,"MEDI"))<br>AND (LIMIT-TO (LANGUAGE,"English"))	

Supplementary table 5: Preferred Reporting Items for Systematic reviews and Meta-Analyses extension for Scoping Reviews (PRISMA-ScR) Checklist (Scoping review 2)

SECTION	ITEM	PRISMA-ScR CHECKLIST ITEM	REPORTED ON PAGE #
**TITLE**			
Title	1	Identify the report as a scoping review.	na
**ABSTRACT**			
Structured summary	2	Provide a structured summary that includes (as applicable): background, objectives, eligibility criteria, sources of evidence, charting methods, results, and conclusions that relate to the review questions and objectives.	2-3
**INTRODUCTION**			
Rationale	3	Describe the rationale for the review in the context of what is already known. Explain why the review questions/objectives lend themselves to a scoping review approach.	5-6
Objectives	4	Provide an explicit statement of the questions and objectives being addressed with reference to their key elements (e.g., population or participants, concepts, and context) or other relevant key elements used to conceptualize the review questions and/or objectives.	8
**METHODS**			
Protocol and registration	5	Indicate whether a review protocol exists; state if and where it can be accessed (e.g., a Web address); and if available, provide registration information, including the registration number.	9
Eligibility criteria	6	Specify characteristics of the sources of evidence used as eligibility criteria (e.g., years considered, language, and publication status), and provide a rationale.	8
Information sources*	7	Describe all information sources in the search (e.g., databases with dates of coverage and contact with authors to identify additional sources), as well as the date the most recent search was executed.	8
Search	8	Present the full electronic search strategy for at least 1 database, including any limits used, such that it could be repeated.	Supp page 7-9
Selection of sources of evidence†	9	State the process for selecting sources of evidence (i.e., screening and eligibility) included in the scoping review.	8
Data charting process‡	10	Describe the methods of charting data from the included sources of evidence (e.g., calibrated forms or forms that have been tested by the team before their use, and whether data charting was done independently or in duplicate) and any processes for obtaining and confirming data from investigators.	8
Data items	11	List and define all variables for which data were sought and any assumptions and simplifications made.	8
Critical appraisal of individual sources of evidence§	12	If done, provide a rationale for conducting a critical appraisal of included sources of evidence; describe the methods used and how this information was used in any data synthesis (if appropriate).	n/a
Synthesis of results	13	Describe the methods of handling and summarizing the data that were charted.	8
**RESULTS**			
Selection of sources of evidence	14	Give numbers of sources of evidence screened, assessed for eligibility, and included in the review, with reasons	Supp page 13

SECTION	ITEM	PRISMA-ScR CHECKLIST ITEM	REPORTED ON PAGE #
		for exclusions at each stage, ideally using a flow diagram.	
Characteristics of sources of evidence	15	For each source of evidence, present characteristics for which data were charted and provide the citations.	14
Critical appraisal within sources of evidence	16	If done, present data on critical appraisal of included sources of evidence (see item 12).	n/a
Results of individual sources of evidence	17	For each included source of evidence, present the relevant data that were charted that relate to the review questions and objectives.	Supp page 14-17
Synthesis of results	18	Summarize and/or present the charting results as they relate to the review questions and objectives.	Supp page 14-17
**DISCUSSION**			
Summary of evidence	19	Summarize the main results (including an overview of concepts, themes, and types of evidence available), link to the review questions and objectives, and consider the relevance to key groups.	18-19
Limitations	20	Discuss the limitations of the scoping review process.	20
Conclusions	21	Provide a general interpretation of the results with respect to the review questions and objectives, as well as potential implications and/or next steps.	20
**FUNDING**			
Funding	22	Describe sources of funding for the included sources of evidence, as well as sources of funding for the scoping review. Describe the role of the funders of the scoping review.	21

Supplementary Figure 1: PRISMA flowchart for Scoping review 1 "What technologies have been described for language mapping during awake craniotomy?"

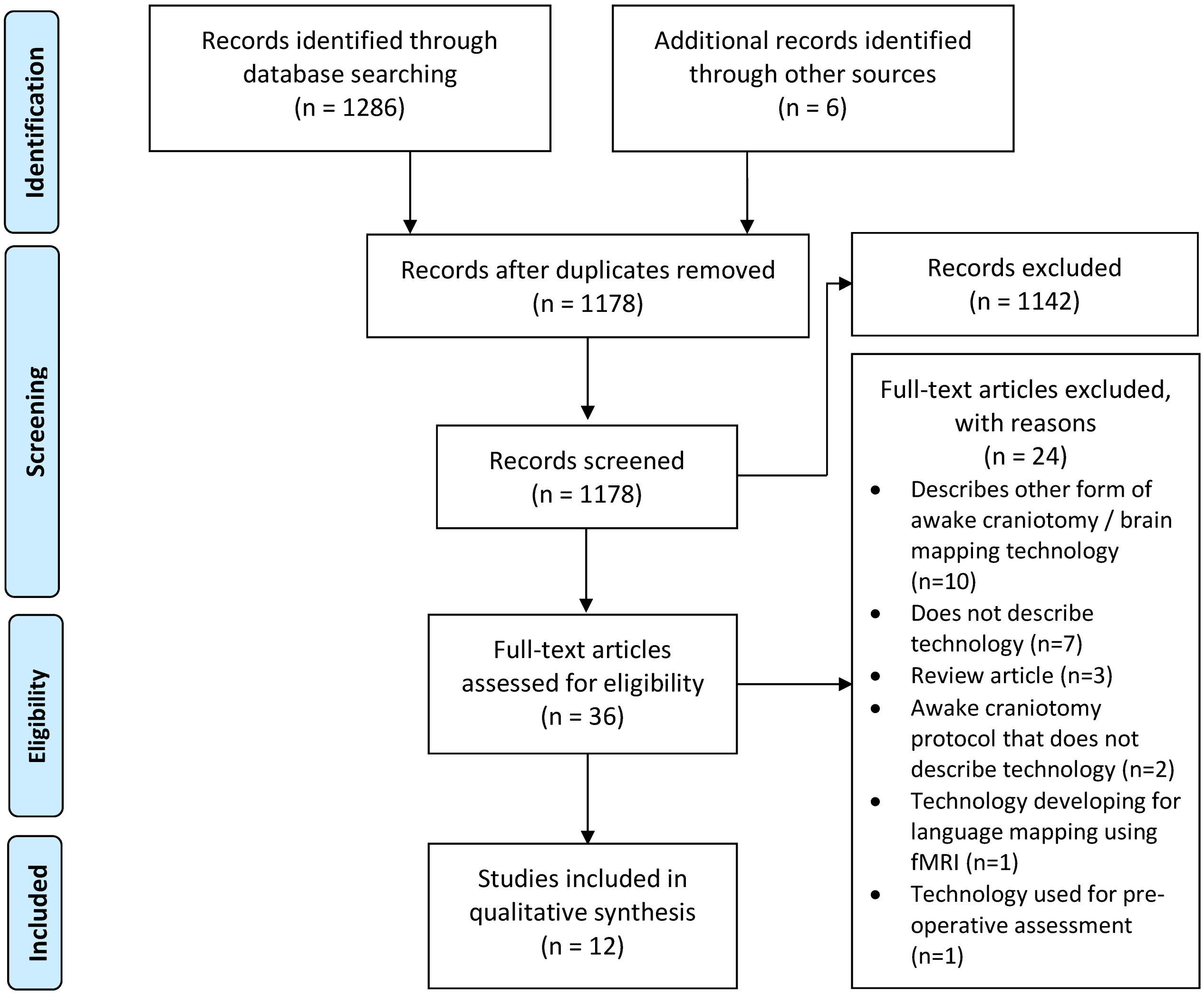

Supplementary Figure 2: PRISMA flowchart for scoping review 2: "What are the barriers and facilitators to adopting novel technology in surgery?"

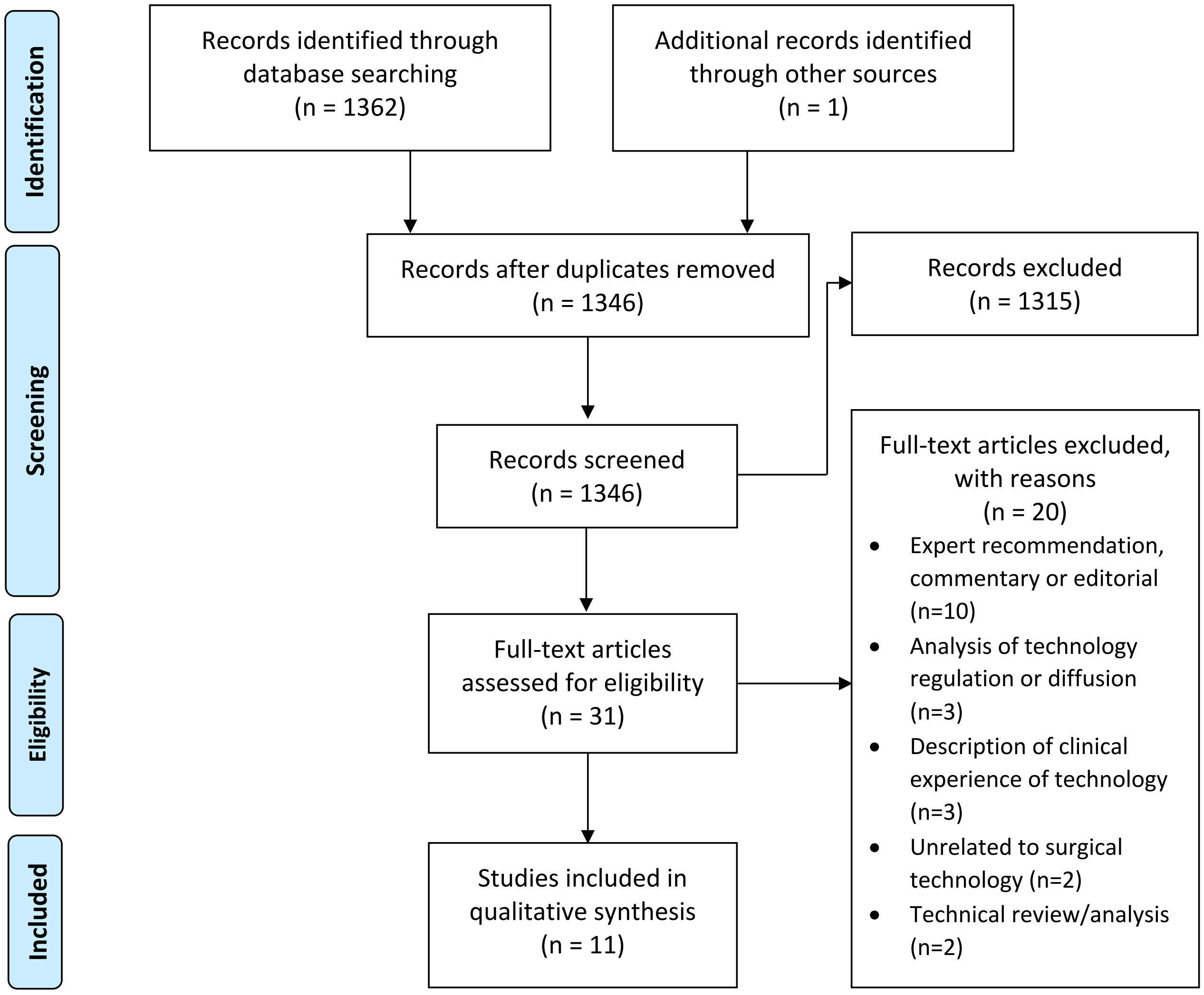

Supplementary Table 6: barriers and facilitators to adoption of novel technology by surgical teams mapped onto the UTAUT constructs

	Facilitator	Barrier
**Performance expectancy**	• Perceived intraoperative benefits to surgeons in terms of visualisation, precision, dexterity (30,31,33–35)<br>• Perceived benefit to patient outcomes (30,31,33,36,37)<br>• Potential for minimising human error (31,34,35)<br>• More ergonomic, which may prolong the working life of surgeons (31,34,36,38)<br>• Increased reliability and consistency (31)<br>• Reduced operative time (31)<br>• Reduced postoperative complications such as infection, bleeding (31,33,36)<br>• Shorter inpatient stay (33,36)<br>• Limited treatment alternatives (37)<br>• Durability (37)<br>• Expectations for further development potential of the technology (31,39)<br>• New layout in the intraoperative environment may increase engagement members of the surgical team (36)<br>• Opinion that technological progress is more important than superiority of performance (38)<br>• Favours minimally invasive surgical approaches (36,38)	• Belief that existing technology fulfils needs sufficiently (30,37)<br>• Lack of trust in performance of the innovation (30)<br>• Lack of knowledge about existing innovations (30)<br>• Lack of haptic feedback in robotic surgery (31,38–40)<br>• Cognitive demands of intraoperative troubleshooting may have a negative impact on overall performance and surgical outcomes (40)<br>• Inexperience costs time intraoperatively (40)<br>• Presents new set of ergonomic issues (38)<br>• Belief that innovation may increase postoperative complications (31)<br>• Perception that patients are subject to increased risk in early period of using innovation (31,33)<br>• Uncertainty about overall benefit (36–38)<br>• View that performance of innovation is still operator-dependent (38)<br>• Difficult to assess superiority of innovation due to limited or biased evidence (37,38)<br>• Performance may vary depending on procedure type (38)
**Effort expectancy**	• Perception that overall outcome is worth it for the patient despite the expected effort required for adoption (30)<br>• May decrease need for operating room “manpower” (39)	• Association of new technologies with increased complexity of use (30)<br>• Technology adoption leads to increased total operative duration (30,31)<br>• The requirement of training programmes (30)

Veljanoski D, *et al. BMJ Surg Interv Health Technologies* 2024; 6:e000234. doi: 10.1136/bmjsit-2023-000234

	• Belief that it will make surgery easier for surgeons (30)<br>• Innovation may shorten the learning curve for procedures (34,39)<br>• Shorter learning curve correlates to increased effectiveness (35,37)<br>• Preoperative planning to minimise disruption to surgery (40)<br>• Simple to use (31)<br>• Experience with using similar pre-existing systems (35)<br>• Effort decreases as experience increases (31,35)	• Requires new set of manual skills for surgeons as well as supporting staff (40)<br>• Requires maintenance and development of innovation-specific skillset (40)<br>• Changes in operating team working environment (for example in robotic surgery surgeons may work at a separate console) (38–40)<br>• Prospect of encountering technical difficulties that are difficult for an inexperienced user to diagnose and fix (40)<br>• Steep learning curve (31)<br>• A lot of effort for limited patient volume if only utilisable for specific procedures (31)
**Social influence**	• Demand from patients (31,36–38)<br>• Exposure in the media leading to increased market drive (31,36)<br>• Prestige and reputation (31,35–37)<br>• Appearances of keeping up with peer groups (31,36,37)<br>• Perception that peers would encourage adoption of innovation (35)<br>• Pressure to be a pioneer in adoption of innovation (36,37)<br>• Approval from diverse groups (37)<br>• Already accepted by other clinicians or institutions (32,37)<br>• “Blind faith”- confirmation bias that innovation correlates to better outcomes independent of corroboration with evidence (36,38)<br>• “gadget-aholics” personality trait (37,38)	• Belief that technology will have dehumanising impact upon relationships between healthcare staff and patients (30)<br>• Perception that adoption of innovation is for individualistic purposes such as personal interest, rather than for benefit of the wider community (30)<br>• Perception of “luxury” and “unnecessary” (30)<br>• Confirmation bias of non-users (38)<br>• Perception that innovations are used as a marketing strategy to attract patients and that this outweighs the actual potential benefit (31)<br>• Ethical issues surrounding inequalities in accessibility to services offering use of surgical innovations (33,39)

	• Academic interest in technology (36,37)	
**Facilitating conditions**	• The assignment of an 'innovation facilitator' role who is responsible for facilitating the progress of adoption (30)<br>• Provision and accessibility of technical support(31,35,37)<br>• Accessibility of the innovation itself (34)<br>• Provision of instruction, guidance, or training programmes (32,35,36,40)<br>• Educational programmes that extend beyond the initial adoption stage of the innovation (32)<br>• Effective collaboration with hospital administration and management, such as with staffing to ensure appropriately trained staff are co-ordinated on work rotas (32,40)<br>• Reasonably priced (37)<br>• Positive cost-benefit ratio and attractive financing options (37)<br>• Sufficient patient cohort (37)<br>• Size of provider- perception that larger hospitals may be more likely to adopt innovations (37)<br>• Effective promotion and marketing from representatives (35,36,38)<br>• Responsibility for decision to adopt innovation is held by management(38)<br>• Market orientated healthcare system (32,38)<br>• Multistakeholder appraisal and collective decision-making approach involving management and clinical teams, rather than the traditional top-down approach in many hospitals whereby management are	• Perception of limited resource (30,33,38)<br>• Systemic resistance to change (30)<br>• Lack of forum to voice ideas or feedback (30)<br>• Lack of encouragement with regards to initiation of innovation(30,31)<br>• Perceived high expenditure in terms of purchase, maintenance and consumables (32,33,38,39)<br>• Low cost-benefit ratio (31,38)<br>• Belief that hospitals need to "ex-novate" by removing out-of-date innovations to free up space for innovation (30)<br>• Belief that organisations will not monitor or evaluate the functioning of innovations sufficiently (30)<br>• Financing options specific to innovations are complicated and inflexible (37)<br>• Requirements for approval varies across countries and institutions (37)<br>• Perceived lack of support from hospital administration (32)<br>• Monopoly market due to limited manufacturers therefore costs stay high (39)<br>• Limited availability of specialist environments that can facilitate innovations (40)<br>• Top-down implementation of decision-making may cause a disregard for the opinions or recommendations of innovation users (40)<br>• Lack of standardisation in innovation training programmes (32)<br>• Requirements for receiving accreditation for innovation may be difficult to fulfil (32)

	the first port-of-call (33,38,40)	
